# Supplementary material for: The causal relationship between the human gut microbiota and pyogenic arthritis: a Mendelian randomization study
Source: Front Cell Infect Microbiol. 2024 Nov 26;14:1452480. doi: 10.3389/fcimb.2024.1452480 (PMC11629706; doi:10.3389/fcimb.2024.1452480)
Supplement: Supplementary file 1 [file DataSheet1.zip › mendelian test/ebi-a-GCST90027712.csv_leave_one_out.pdf]

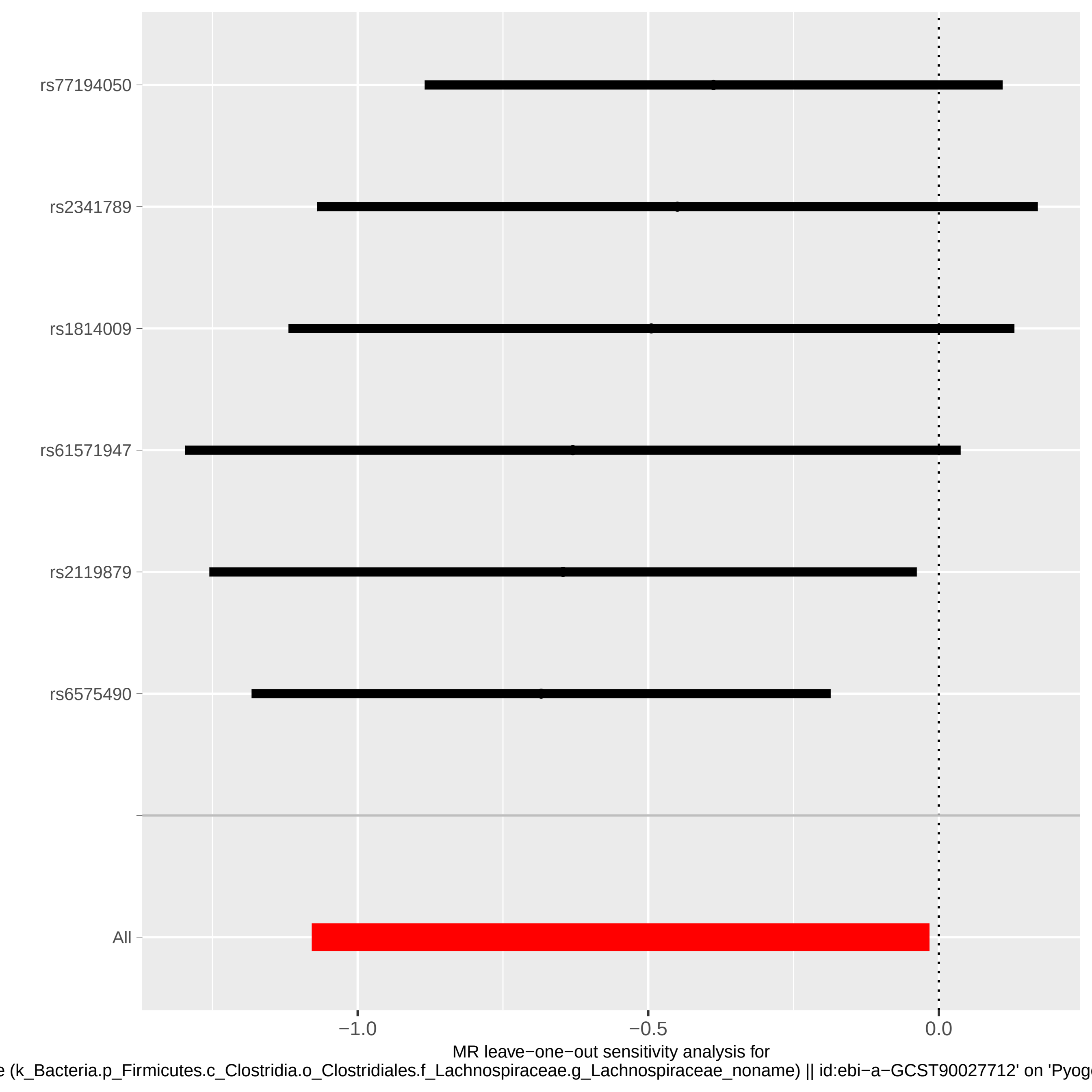
rs77194050
rs2341789
rs1814009
rs61571947
rs2119879
rs6575490
All
−1.0
−0.5
0.0
MR leave−one−out sensitivity analysis for
e (k_Bacteria.p_Firmicutes.c_Clostridia.o_Clostridiales.f_Lachnospiraceae.g_Lachnospiraceae_noname) || id:ebi−a−GCST90027712' on 'Pyog
